# Multifocality and Multicentrality in Breast Cancer: Comparison of the Efficiency of Mammography, Contrast-Enhanced Spectral Mammography, and Magnetic Resonance Imaging in a Group of Patients with Primarily Operable Breast Cancer

**DOI:** 10.3390/curroncol28050341

**Published:** 2021-10-08

**Authors:** Katarzyna Steinhof-Radwańska, Andrzej Lorek, Michał Holecki, Anna Barczyk-Gutkowska, Anna Grażyńska, Joanna Szczudło-Chraścina, Oskar Bożek, Justyna Habas, Karol Szyluk, Paweł Niemiec, Iwona Gisterek

**Affiliations:** 1 Department of Radiology and Nuclear Medicine, Prof. Kornel Gibiński Independent Public Central Clinical Hospital, Medical University of Silesia in Katowice, 40-752 Katowice, Poland; anna.barczyk@sum.edu.pl (A.B.-G.); oskar.bozek@sum.edu.pl (O.B.); 2 Department of Oncological Surgery, Prof. Kornel Gibiński Independent Public Central Clinical Hospital, Medical University of Silesia in Katowice, 40-514 Katowice, Poland; alorek@sum.edu.pl; 3 Department of Internal, Autoimmune and Metabolic Diseases, Faculty of Medical Science, Medical University of Silesia, 40-752 Katowice, Poland; mholecki@sum.edu.pl; 4 Students’ Scientific Society Department of Nuclear Medicine and Diagnostic Imaging, Faculty of Medical Sciences in Katowice, Medical University of Silesia in Katowice, University Clinical Center Prof. K. Gibiński, 40-752 Katowice, Poland; grazynska.anna@gmail.com; 5 Department of Diagnostic Imaging, Oncology Hospital, 43-300 Bielsko-Biała, Poland; jszczudlo@onkologia.bielsko.pl; 6 Faculty of Pharmaceutical Sciences, Medical University of Silesia in Sosnowiec, 41-200 Sosnowiec, Poland; justyna.habas@gmail.com; 7 I Department of Orthopaedic and Trauma Surgery, District Hospital of Orthopaedics and Trauma Surgery, 41-940 Piekary Śląskie, Poland; szyluk@urazowka.piekary.pl; 8 Department of Physiotherapy, Faculty of Health Sciences in Katowice, Medical University of Silesia in Katowice, 40-752 Katowice, Poland; 9 Department of Biochemistry and Medical Genetics, Faculty of Health Sciences in Katowice, Medical University of Silesia in Katowice, 40-752 Katowice, Poland; pniemiec@sum.edu.pl; 10 Department of Oncology and Radiotherapy, Prof. Kornel Gibiński Independent Public Central Clinical Hospital, Medical University of Silesia in Katowice, 40-515 Katowice, Poland; igisterek@sum.edu.pl

**Keywords:** breast cancer, contrast-enhanced spectral mammography, mammography, comparative studies, pathology, surgery, multifocal and multicentral breast cancers

## Abstract

Background: The multifocality and multicentrality of breast cancer (MFMCC) are the significant aspects that determine a specialist’s choice between applying breast-conserving therapy (BCT) or performing a mastectomy. This study aimed to assess the usefulness of mammography (MG), contrast-enhanced spectral mammography (CESM), and magnetic resonance imaging (MRI) in women diagnosed with breast cancer before qualifying for surgical intervention to visualize other (additional) cancer foci. Methods: The study included 60 breast cancer cases out of 630 patients initially who underwent surgery due to breast cancer from January 2015 to April 2019. MG, CESM, and MRI were compared with each other in terms of the presence of MFMCC and assessed for compliance with the postoperative histopathological examination (HP). Results: Histopathological examination confirmed the presence of MFMCC in 33/60 (55%) patients. The sensitivity of MG in detecting MFMCC was 50%, and its specificity was 95.83%. For CESM, the sensitivity was 85.29%, and the specificity was 96.15%. For MRI, all the above-mentioned parameters were higher as follows: sensitivity—91.18%; specificity—92.31%. Conclusions: In patients with MFMCC, both CESM and MRI are highly sensitive in the detection of additional cancer foci. Both CESM and MRI change the extent of surgical intervention in every fourth patient.

## 1. Introduction

Breast cancer is the leading cause of death in women worldwide, manifesting the most frequent malignancy [1]. The choice of local and systemic treatment for breast cancer depends on both the histological type and grading, the progression of the primary tumor, the status of regional lymph nodes, and the presence of metastases, as well as the coexistence of other diseases and individual patient preferences [2,3]. Multifocal and multicentric breast cancers (MFMCC) may be the significant factors influencing a surgeon’s choice between breast-conserving therapy (BCT) and mastectomy [4,5]. Multifocal breast cancer, when at least two invasive tumors develop in the same quadrant, or area, of the breast. All tumors arise from one original tumor. Multicentric breast cancer is where at least two tumors develop separately, often in different areas of the breast [6,7].

Among many attainable imaging methods, mammography is the basic method for detecting neoplastic lesions in the breast, as this method is relatively inexpensive, widely available, and reproducible. The sensitivity of mammography (MG) depends on the structure of the breast. It should be taken into account that it decreases and ranges from 45% to approximately 60% in breasts, with a predominance of glandular tissue [8,9].

Magnetic resonance imaging (MRI) is based on the presence of increased neoangiogenesis in the tumor. Thus, this examination provides information about the morphology of the focal lesions but also an analysis of kinetics and dynamics of contrast enhancement. MRI extended with diffusion-weighted imaging/apparent diffusion coefficient (DWI/ADC) is a test with a high level of sensitivity and specificity (over 85%) [10,11]. MRI is a method that has not been used in the first-line diagnosis of breast cancer; however, due to its high sensitivity and specificity, it is widely used in high-risk patients (e.g., *BRCA 1* and *BRCA 2* mutations carriers). In these patients, compared with MG, MRI offers a doubled or even tripled sensitivity. MRI is also an extremely useful method in patients with lobular cancer and in patients who have increased breast density. In these women, the effectiveness of MG is underestimated, and there is a risk of missing neoplastic lesions. It is recommended that women with increased breast density be offered systematic abbreviated MRI for screening. MRI also finds application in monitoring responses to neoadjuvant chemotherapy, excluding multifocality and multicentrality of breast cancers, as well as searching for the primary origin lesion in the case of axillary nodal metastases [12]. Contrary to mammography, which underestimates the tumor size, which can result in incomplete resection, MRI is more accurate in imaging the local extent of breast cancer, tumor size, and carcinoma location. Furthermore, some carcinomas and foci are seen only on breast MRI scans [13]. It is worth considering the limitations of MRI, the first of which is the unsatisfactory rate of false-positive (FP) results. False-positive results may result in more aggressive therapeutic management than obligatory treatment, which, in turn, affects the patient’s quality of life. The main causes of FP results include non-mass-like enhancement, mastopathic changes, fibrocystic changes due to hormonal stimulation, inflammatory changes for benign lesions or ductal carcinoma in situ (DCIS), invasive lobular carcinoma, and some cases of estrogen receptor-negative invasive ductal carcinoma [14].

Moreover, microcalcifications are not detectable, and the acquisition time is quite long, ranging approximately from 20 to 30 min [15,16]. It is worth noting that several types of premalignant neoplasms increase the risk of breast carcinomas, such as lobular carcinoma in situ, intraepithelial neoplasia, and different atypias, and atypical ductal hyperplasia and atypical lobular hyperplasia are responsible for false-positive lesions. The aforementioned aspects should be taken into account while discussing the percentage of FP results in MRI. Recently created new MRI protocols, called fast protocols, provide significantly shorter scanning time, along with comparable efficacy to conventional full MRI examination [17,18].

Contrast-enhanced spectral mammography (CESM) is a new technique, intensively developed in recent years and accepted by the FDA for clinical use in the United States in 2011. This method, as with MRI, is based on imaging of tumor neoangiogenesis by use of a contrast agent (chelated iodine-based X-ray contrast agent) [19,20]. CESM is based on the double-energy technology that capitalizes on the inherent difference in X-ray attenuation of breast tissue and iodine. It provides morphological information accessible in conventional mammography and additionally makes it possible to visualize breast areas that exhibit the enhanced uptake of the contrast agent most commonly related to neoangiogenesis, as is the case with breast MRI. CESM uses X-rays similar to conventional mammography. The average glandular dose (AGD) for a low-energy image is equal to one conventional mammography, while for a high-energy image, it is approximately 20% of the dose from one conventional mammography. The sensitivity of CESM in detecting breast cancer is over 90% [21]. As shown in numerous studies, CESM is significantly more sensitive than standard mammography in detecting breast cancer in patients with dense breasts and is independent of the patients’ menopausal status. In addition, it has a much lower FP rate than MRI. Only a few studies have evaluated bilateral CESM as an alternative to MRI, indicating its analogous capability to depict lesion morphologic features and perfusion characteristics while achieving them at a minor cost and with faster image acquisition. The sensitivity of both CESM and MRI was reported to be over 90% [21,22,23,24]. Although the sensitivity of CESM and MRI is high for both techniques, the positive predictive value (PPV) was reported to be higher for CESM than MRI (60% vs. 93%, respectively) [24]. In another study, it was reported that the difference between PPVs for both methods was not that high anymore, with 90.5% for MRI and 94.7% for CESM [25]. As previously mentioned, the difference in PPVs may be due to the higher number of FP scores with MRI than with CESM. Moreover, it is not without significance that the specificity of CESM is higher than MRI (84% vs. 80.2%, respectively) [25,26].

It is worth noting that multicentral carcinomas are more common in young patients or perimenopausal women with large tumors (>5 cm) and high-density fibroglandular parenchyma, as well as in women with a family history of breast cancer [27]. Similarly, in cases of invasive lobular carcinoma, multicentral carcinomas may occur [28]. Therefore, breast cancer detection requires a multimodal approach, and the radiologist must apply several imaging modalities appropriately.

The objective of the study was to assess the usefulness of MG, CESM, and MRI in women diagnosed with breast cancer before qualifying for surgical intervention to visualize other (additional) cancer foci. The second aim of our work was to evaluate the difference in surgical decisions made upon the application of CESM and MRI versus those based on MG.

## 2. Materials and Methods

**Figure 1:**
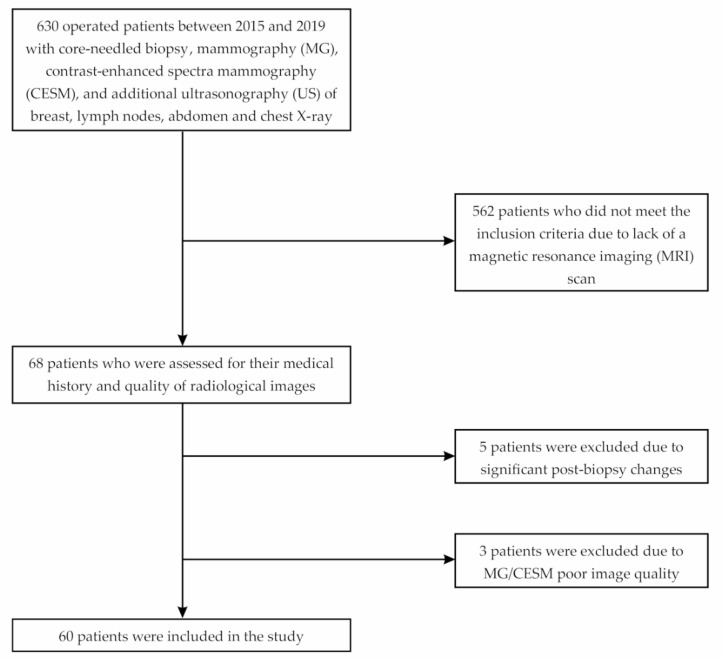
Flowchart of the study.

In this study, we analyzed the medical records of 630 patients with primary operable breast cancer. The surgeries were performed between January 2015 and April 2019 at the Department of Oncological Surgery, University Clinical Center of the Medical University of Silesia in Katowice, Poland. The subjects were included in the analysis following crucial inclusion criteria, such as histopathologically confirmed breast cancer based on samples collected by core needle biopsy and a completed set of radiological diagnostics, i.e., digital mammography, contrast-enhanced spectral mammography, and magnetic resonance imaging. When qualifying for surgical treatment, the following factors were taken into account: tumor size, presence of additional lesions, and the patient’s preferences. The criterion excluding sparing treatment was the inability to obtain a satisfactory aesthetic effect. We excluded patients with significant postbiopsy changes (e.g., hemorrhage) affecting image quality. Among 630 patients, only 60 met the criteria and were allowed to proceed with the retrospective analysis (Figure 1).

Ethical committee approval was not required due to the retrospective nature of the study and the lack of criterion of a medical experiment (Bioethics Commission Decision PCN/0022/KB/75/20, 15 May 2020). All the test procedures were carried out in compliance with the ethical principles of the 1964 Helsinki Declaration and its subsequent amendments.

### 2.1. Diagnostics 

Our department deals with comprehensive diagnostics and treatment of breast cancer, so patients from other facilities are referred to our hospital. All the subjects had an MG examination that was performed outside our department. However, each of the obtained results of MG was estimated again by two of our specialists with a total work experience of over 20 years. Afterward, the patients underwent a CESM examination, which is performed in our facility in every patient with a breast carcinoma before qualification for surgery treatment (after giving informed consent and the verification of no contraindications for the examination).Absolute contraindications to performing the CESM examination are as follows: impaired renal function with chronic kidney disease; an estimated glomerular filtration rate (eGRF) less than 30 mL/min; a history of an anaphylactic or anaphylactoid reaction to iodinated contrast agents; pregnancy; a known BRCA1 or BRCA2 mutation due to radioprotection; known hyperthyroidism.

MRI examination was not performed on every subject in accordance with current European Society of Breast Cancer Specialists (EUSOMA) guidelines according to which MRI is performed on patients diagnosed with lobular carcinoma and on patients under 60 years of age with a tumor size difference of >1 cm between MG and ultrasonography (US) examination and when this difference could determine the type of surgical procedure [29,30]. MRI examinations were performed in patients meeting the EUSOMA criteria.

Moreover, US examinations of the breast, lymph nodes, and abdomen were performed in each of the patients to detect possible metastases. After completing the diagnostics, the final therapeutic decision was made based on an arrangement of interdisciplinary case conference of the Breast Cancer Unit (BCU) with the participation of the patient and a team of specialists, including an oncological surgeon, a clinical oncologist, a radiotherapist, a radiologist, and a pathomorphologist. The patient was able to ask questions and expressed informed consent to the proposed treatment. After consultations, the patients underwent surgeries. 

### 2.2. Imaging Procedures

**Figure 2:**
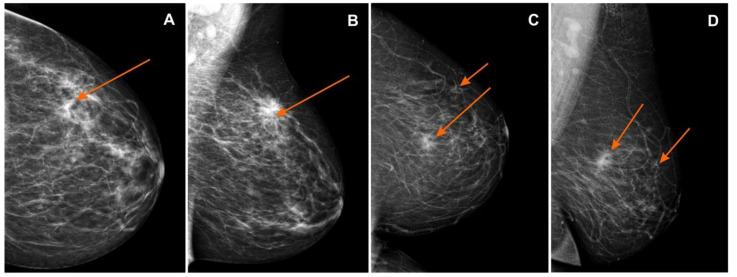
Mammography (MG): (**A**) craniocaudal (CC) and (**B**) mediolateral oblique (MLO) views—pathological mass in the left breast in the superior-outer quadrant (orange arrows), invasive lobular carcinoma GII, BI-RADS 6; (**C**) craniocaudal (CC) and (**D**) mediolateral oblique (MLO) views—pathological mass in the left breast in the superior-inner quadrant (orange arrows), infiltrating duct carcinoma, BI-RADS 6. BI-RADS: Breast Imaging-Reporting and Data System.

**Figure 3:**
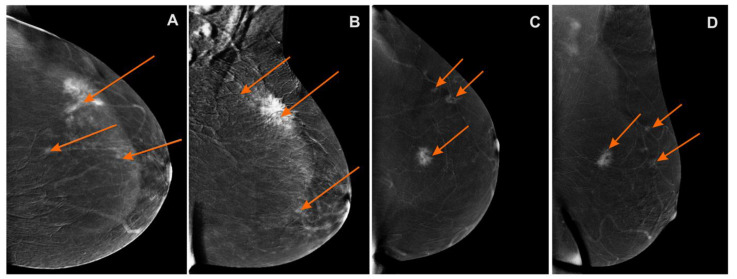
Contrast-enhanced spectral mammography (CESM)subtraction images: (**A**) craniocaudal (CC) and (**B**) mediolateral oblique (MLO) views—irregular mass with heterogeneous enhancement and long spiculated mass BI-RADS 6. Numerous enhanced small foci are noted in left breast BI-RADS 4 (orange arrows); (**C**) craniocaudal (CC) and (**D**) mediolateral oblique (MLO) views—irregular mass with heterogeneous enhancement and long spiculated mass BI-RADS 6. Additional enhanced small foci are noted in the left breast in superior-outer quadrant BI-RADS 4 (orange arrows).

**Figure 4:**
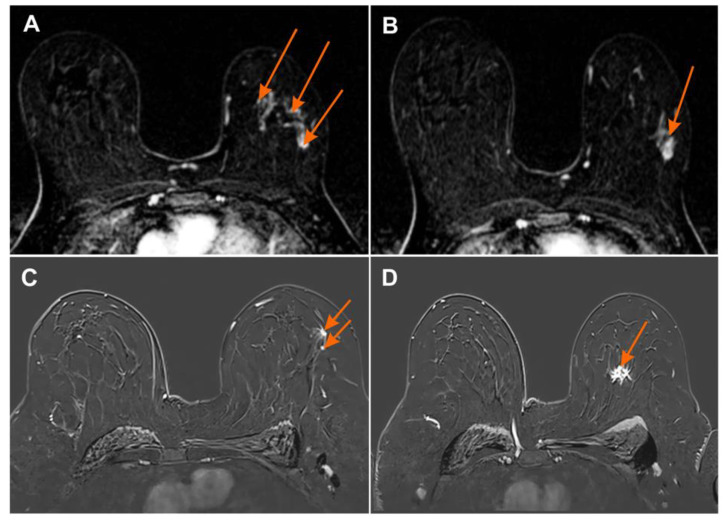
Magnetic resonance imaging (MRI) subtraction image (**A**,**B**) 2 min after contrast injection irregular mass with heterogeneous enhancement BI-RADS 6 with numerous foci of contrast uptake showing multicentric cancer BI-RADS 4 (orange arrows); (**C**,**D**) MRI subtraction image 2 min after contrast injection irregular mass with heterogeneous enhancement BI-RADS 6 with additional foci of contrast uptake showing multicentric cancer BI-RADS 4 (orange arrows).

The MG, CESM, and MR images were assessed based on the Breast Imaging-Reporting and Data System (BI-RADS, according to ACR BI-RADS Atlas® 5th Edition) [31]. A lesion that had already been confirmed to be cancerous in core-needle biopsy was classified as BI-RADS 6, while additional foci suspected of the multifocal or multicentric neoplastic process were classified as BI-RADS 4 or BI-RADS 5. (Figure 2, Figure 3 and Figure 4). Next, the additionally identified lesions (suspected MFMCC) were visualized on second-look ultrasound or in MRI and subjected to core-needle biopsy (under US or MRI). The statistical analysis included one (i.e., the biggest) dimension of the tumor.

### 2.3. Contrast-Enhanced Spectral Mammography (CESM) Protocol

All CESM examinations were carried out with a digital mammography device dedicated to performing dual-energy CESM acquisitions (SenoBright, GE Healthcare, Waukesha, WI, United States). An intravenous injection of 1.5 mL/kg of body mass of non-ionic contrast agent was performed using a power injector at a rate of 3 mL/s with a bolus chaser of 30 mL of saline. In CESM mode, the device automatically performed a pair of exposures (low and high energy) in each view. Specific image processing of low- and high-energy images was performed to obtain subtraction images to highlight contrast enhancement and suppress structured noise due to fibroglandular breast tissue. The total examination time was usually 10 min. After examination, the patients were observed for approx. 30 min for any adverse reactions that may occur after administration of the contrast agent.

### 2.4. Magnetic Resonance Imaging (MRI) Protocol

All contrast-enhanced MRI examinations were performed with 1.5T and 3T MRI systems (Signa 1.5T, GE Healthcare, Chicago, IL, United States; 3T Magnetom Vida, Siemens Medical Systems, Erlangen, Germany). All patients underwent MRI examination in the prone position using a dedicated 4-channel breast coil (1,5T) and 18-channel breast coil (3T). Our protocol in the transversal plane included the following: T1-weighted gradient-echo sequence (FFE/fl2d), T2-weighted turbo/fast spin-echo (TSE/FSE) with and without fat saturation (thickness of the layer was 3 mm), echo-planar diffusion-weighted imaging with apparent diffusion coefficient (thickness of the layer was 4 and 5 mm), and dynamic 3d sequence with fat saturation was performed (vibrant/fl3d) before the administration of contrast agent, followed by 4–6 repetitions of the same sequence after injection. The duration of each postcontrast acquisition was 60 to 90s, depending on breast size, and the slice thickness was less than or equal to 2 mm. Postcontrast dynamic MR images were acquired after the administration of 0.1 mmol/kg of body mass gadolinium contrast agent.

### 2.5. Histopathological Examination

The histopathological (HP) examination was performed in the Histopathology Laboratory of our center by 2 pathologists with extensive experience (of more than 15 years) in breast cancer diagnostics. The greatest dimension of the tumor necessary for determining the T descriptor in the pathological TNM (pTNM) classification, besides the macroscopic measurement, was verified histopathologically using a microscope and OlympuscellSens Dimension® software (Tokyo, Japan). Tumors up to 2 cm were excised in whole, serially, on a cross-sectional basis with a margin of 0.2 to 0.4 cm and embedded in a paraffin block, after each cross section. Tumors measuring over 2 cm, not fitting within a single paraffin block, were divided into 2 or more parts by making parallel cuts of the lesion. Next, they were marked in pairs with the ink of the same color, and the individual layers were given numbers to allow the restoration of the entire largest section of the tumor. The T value of the tumor was the total of the parallel measurements of the particular parts of the lesion.

The tumors were defined as MFMCC if two lesions were separated by at least 5 mm of healthy tissue. The histological features of all the additional neoplastic foci diagnosed histopathologically were defined, including the tumor’s size, type, and malignancy level. The study included invasive cancers and in situ cancers.

### 2.6. Data Analysis and Statistical Method

The analysis included the results of 60 patients selected according to the above-mentioned inclusion criteria. Patients’ age distribution was analyzed and tested for normality using the Kołmogorov–Smirnov test. The average, minimum and maximum values in the sample, as well as standard deviation, were determined for the variable studied. The subsequent part of the statistical analysis involved the construction of contingency tables for the results of MFMCC detectability for each of the diagnostic methods under analysis, compared with HP. The analysis of these tables served as the basis for calculating the values of sensitivity, specificity, negative predictive value (NPV), and positive predictive value for each of the methods (MG, CESM, and MRI). The 95% confidence intervals for the calculated sensitivity and specificity values were determined based on the Clopper–Pearson estimation method, using the Z test for a single proportion. Next, graphs of receiver operating characteristic (ROC) curves were drawn up for each of the methods, and the values of the areas under the curve (AUC) field were calculated and compared with each other. Standard errors were also calculated for half-AUC. The significance limit for the calculations was established at *p* = 0.05. A quantitative summary was also prepared for the histopathological types of cancers, and the level of their detectability was determined for the diagnostic methods under analysis. The diagnostic results in the methods under analysis served as the basis for determining the rate of decision change in the treatment procedure. The data were analyzed using a Microsoft Office Excel 2013 spreadsheet (Microsoft Corporation, Redmond, WA, United States) and the Statistica software (STATISTICA 13.1, StatSoft Inc., Tulsa, OK, United States).

## 3. Results

Patients’ median age was 62 years (range 26–86 years). Histopathological examination confirmed the presence of MFMCC in 33/60 (55%) patients. The sensitivity of MG in detecting MFMCC was 50%, and its specificity, PPV, and NPV were 95.83%, 94.44%, and 57.5%, respectively. For CESM, the sensitivity was 85.29%, and its specificity, PVV, and NPV were 96.15%, 96.67%, and 83.33%, respectively. For MRI, all above-mentioned parameters were higher, as follows: sensitivity—91.18%; specificity—92.31%; PPV—93.94%; NPV—88.89%. The consistency of histopathological examination with the results of MG, CESM, and MRI in terms of detecting MFMCC is presented in Table 1.

**Figure 5:**
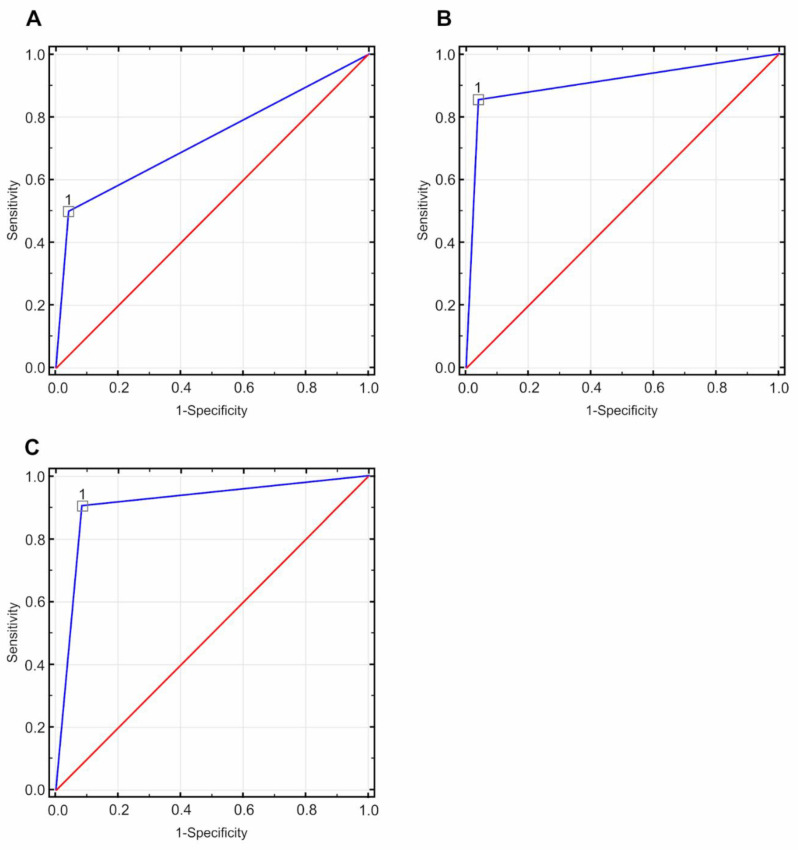
Receiver operating characteristic (ROC) curves based on the tested diagnostic methods (directional coefficient = 1.00, suggested cut-off point = 1.00): (**A**) for the mammography (MG) method, the value of the area under the curve (AUC) field was 0.729 with a standard error of 0.066, *p* < 0.05; (**B**) for the contrast-enhanced spectral mammography (CESM) method, the value of the AUC field was 0.907 with a standard error of 0.042, *p* < 0.05; (**C**) for the magnetic resonance imaging(MRI) method, the value of the AUC field was 0.917, with a standard error of 0.042, *p* < 0.05.

The ROC curves were determined based on MG, CESM, and MRI results (Figure 5). 

Results of the verification of which histopathological types of cancers are detected as multifocal in comparable examination techniques are presented in Table 2. 

The number of procedures performed in the group of 60 patients under analysis is as follows: 34 (56%) breast-conserving surgeries and 26 (43%) different types of mastectomy procedures. The most common type of breast-conserving surgery was wide local excision (WLE) with sentinel lymph node biopsy (SLNB) (25/60, 43.3%). The other types were wide local excision (WLE) with axillary lymph node dissection (ALND) (6/60, 10%) and WLE (2/60, 3.3%).

Based on the MG performed, we planned to conduct 42 conserving surgeries (WLE) in the study group. Upon visualizing 12 cases of MFMCC following CESM and MRI, a decision was taken to conduct different types of mastectomy procedures. The decision change rate was 12/42 = 28.5%.

In those 12 patients, the HP results confirmed MFMCC in 11 (91.6%) cases. In one (8.4%) case, the results obtained were false positive (the preoperative core needle biopsy revealed atypical ductal hyperplasia). In the group of MFMCC patients on conserving therapy, there were positive margins (R1 resection) in one case in HP examination, which required local radicalization.

The analysis involved the number of changes in the extent of conserving treatment into different mastectomies upon the identification of MFMCC in CESM and MRI (Table 3). 

## 4. Discussion

Most of the research released in recent years concentrates on comparing the sensitivity and specificity of CESM and MRI in their ability to detect singular breast cancer sites, and with that, omitting additional neoplastic sites that affect the scope of surgical procedures [32,33]. The leading interest of the present analysis is the multifocality and multicentrality of breast cancer and the impact of those additional lesions on therapeutic decisions. 

Literature data suggest that the occurrence of multifocal and multicentric breast cancers varies between 9 and 75%. These numbers differ due to the method of histopathological samples collection or using different imaging modalities. It has to be considered that such inconsistency between authors is a result of a lack of standardization of the MFMCC definition [34,35]. Eventually, a full analysis of every case should rely on a combination of macroscopic and microscopic evaluation of pathological lesions. Tot et al. [36] in their study showed that 40% of breast cancer had simple (unifocal) subgross morphology, while 60% presented complex morphology with multifocal or diffuse components.

In our previous study, which analyzed 999 patients with a diagnosed breast cancer, we proved that the frequency of MFMCC in the histopathological examination was 19.42% [37]. In the case of our current study, the frequency of MFMCC in histopathological examination reached up to 55%. This difference is due to the fact that a specific group of female patients qualified for this study, which results in a small population of the studied patients. In our center, during the qualification of patients for the MRI examination, we followed the EUSOMA [29,30] criteria, which were not fulfilled by every female patient. With the intention of comparing the effectiveness of CESM and MRI in MFMCC evaluation, we had to exclude all patients without MRI examination, which largely reduced the study group. Due to the group specificity fulfilling EUSOMA recommendations, the number of MFMCCs in histopathology examination is higher than in other research works. However, despite the abovementioned factors, we consider our study valuable, as it shows how CESM performs in detecting MFMCC, compared with MRI. These results should be confirmed by future studies in which the EUSOMA criteria can be enriched by CESM interchangeably with MRI in lesion detection in this group of patients.

Surgery plays an essential role in the treatment of breast cancer. However, it should be noted that precise preoperative knowledge about the extent, size, and location of neoplastic lesions is a requirement for adequate surgical intervention. The basic examinations for proper treatment qualification include mammography and ultrasonography. The sensitivity of digital mammography in detecting multiple breast lesions depends on breast structure. The greater the content of glandular tissue is, the lower the sensitivity of mammography is [38]. However, today, it is unfortunate to state that young women have glandular breasts and elderly women have fatty breasts. Sprague et al., who determined the mammographic breast density distribution by age and body mass in a group of over 1.5 million females, found that heterogeneous or extremely dense breasts were present in 43.3% of women aged 40 to 74 years [39]. In this study, the sensitivity of MG for detecting additional cancer foci was 42%. It seems that the glandular and adipose-gland structures of the breast and the tumor density similar to that of the surrounding glandular tissue resulted in such a low sensitivity in detecting additional cancer foci.

Complementary radiation therapy is required after conserving breast surgery. Irradiation of the entire breast is designed to reduce the rate of local recurrences and eliminate other tumor foci in the mammary gland. For several years, efforts have been made to reduce the volume of irradiated chest tissues to minimize late radiation reactions in the heart and lungs. For this purpose, in early Luminal A cancer in postmenopausal women, accelerated partial breast irradiation (APBI) is used, but when using this technique of radiotherapy, we must exclude the multifocality and multicentricity of cancer [40,41,42].

As it is almost impossible to exclude the presence of additional cancer foci based on either mammography or ultrasonography, it seems reasonable to use imaging techniques with higher sensitivity. MRI has documented higher sensitivity in detecting neoplastic lesions than both MG and US [43,44]. It was testified that even as much as 14–16% of tumors detectable by MRI may remain unseen in MG [45,46].

In our analysis, the use of MRI in preoperative diagnostics resulted in a change in the treatment regimen in 24% of subjects. Our results correspond with data obtained by other researchers—performing an MRI examination in breast cancer patients results in changes in the treatment method in every fifth patient [47]. Despite the evidence of frequent modification of therapeutic decisions after MRI examination in patients with breast cancer, this method remains controversial in this group of patients. In fact, in a multicenter clinical trial, the authors of Comparative Effectiveness of MRI in Breast Cancer (COMICE) did not prove the unequivocal benefits of using MRI in the diagnosis of breast cancer. The subjects in the COMICE trial were mostly postmenopausal women with ACR BI-RADS group 2, and only 9% of them had the luminal type of breast cancer. The authors showed a higher percentage of multifocal and multicenter tumors in the MRI group, but this difference was neither examined nor discussed. Moreover, most reoperations were performed due to the non-radical nature of previously performed ones [48]. 

Furthermore, the authors of the MRI mammography of the Nonpalpable Breast Tumors (MONET) trial did not depict any key benefits of using preoperative MRI examination. This was probably due to the study being aimed at the diagnosis of non-palpable breast tumors in which there are small and very diverse clinical stages of cancers. Notably, patients after MRI examination were characterized by an increased re-excision rate [49]. It should be mentioned, however, that the COMICE trial included centers without the possibility of MRI-guided biopsy. As a result, some of the patients underwent surgery without prior histopathological assessment of the visible foci. Furthermore, the level of radiologists’ experience was much lower than it is now, as this method was relatively new. Finally, there was no standard MRI protocol for all centers.

In our study, the sensitivity of MRI for detecting additional cancer foci was 94.7%. The decision to change the range of surgery from conserving treatment to mastectomy was made in every fourth woman (28.5%), after a core-needle biopsy of the revealed lesion. It is worth noting that the radiologists in our center have at least 20 years of experience in both performing and evaluating MRI; moreover, all suspected foci were additionally verified by core-needle biopsy.

In our analysis, CESM showed high sensitivity (85.29%) and specificity (96.15%) in detecting MFMCC. In their study, Lee-Felker et al. [24] showed that CESM is as sensitive as MRI in detecting additional foci of disease, with CESM identifying 11 of 11 (100%) of additional foci, compared with 10 (91%) with MRI. However, Jochelson et al. [50], in a preoperative examination of breast cancer patients, showed that CESM was less sensitive in detecting additional ipsilateral neoplastic lesions, compared with MRI (CESM detected 14/25 (56%) additional tumors, and MRI 22/25 (88%)). However, both authors suggest that CESM has the potential to be a useful additional imaging method in women with breast cancer when selecting an appropriate surgical method.

According to EUSOMA guidelines, the MRI examination is currently recommended in the following clinical situations: a newly diagnosed lobular breast carcinoma confirmed by breast biopsy, patients with a genetically detected mutation, and patients under the age of 60 years who manifest a discrepancy of more than 1 cm in the tumor size between MG and US [29,30]. On the contrary, there is no recommendation for the use of CESM. It seems to be incomprehensible, as contrast-enhanced spectral mammography is highly sensitive in detecting breast cancer—comparable to that of MRI. Moreover, the tumor’s dimensions in CESM correlate well with histopathological examinations; the cost of CESM is lower than that of MRI; lastly, the time needed to perform and interpret the results is less than with MRI [51]. In our CESM analysis, one patient had false-positive results, but in MRI examination, there were two false-positive ones. However, it should be noted that a preoperative core-needle biopsy revealed atypical intraductal hyperplasia in these cases.

The use of CESM and MRI allows better results to be achieved in the diagnosis of MFMCC, compared with MG, which significantly influences surgical decisions. Precise breast imaging and visualization of additional cancer foci may, in the future, diminish the quantity of postoperative breast radiotherapy after conserving treatment in a much larger group of patients. Such a procedure will permit a reduction in the number of complications in patients, and consequently, it will lower the treatment costs.

Our study has some limitations: mainly, it was performed with a relatively small group of patients, which was due to the restrictive inclusion criteria. This is due to the fact that not all patients diagnosed with breast cancer that qualified for surgery had an MRI scan. MRI was only used in those patients who met EUSOMA recommendations. Due to this specific group of patients, the distribution of tumor types and the incidence of MFMCC may differ from other studies. Other limitations of our study were that it was a single-center study, and all CESM examinations were conducted on a single vendor system. Another limitation of our work is the fact that the MG examinations were conducted outside our center and reassessed by our radiologists.

## 5. Conclusions

In patients with multifocal/multicentral breast cancer who fulfill EUSOMA recommendations, both CESM and MRI are highly sensitive in the detection of additional cancer foci. Both CESM and MRI change the extent of surgical intervention in every fourth patient.

## Figures and Tables

**Table 1:** Compliance of MG, CESM, and MRI in terms of detecting MFMCC, confirmed in postoperative histopathological examination.

Assessment		HP Multifocal	HP Unifocal	
**MG**	multifocal	17	1	PPV 94.44% (95% CI: 72.71–99.86)
	unifocal	17	23	NPV 57.50% (95% CI: 40.89–72.96)
		Sensitivity 50.00% (95% CI: 32.43–67.57)	Specificity 95.83% (95% CI: 78.88–99.89)	
**CESM**	multifocal	29	1	PPV 96.67% (95% CI: 82.78–99.92)
	unifocal	5	25	NPV 83.33% (95% CI: 65.28–94.36)
		Sensitivity 85.29% (95% CI: 68.94–95.05)	Specificity 96.15% (95% CI: 80.36–99.90)	
**MRI**	multifocal	31	2	PPV 93,94% (95% CI: 79.77–99.26)
	unifocal	3	24	NPV 88.89% (95% CI:70.84–97.65)
		Sensitivity 91.18% (95% CI: 76.32–98.14)	Specificity 92.31% (95% CI: 74.87–99.05)	

Abbreviations: MG—mammography; CESM—contrast-enhanced spectral mammography; MRI—magnetic resonance imaging; HP—histopathological examination; CI—confidence interval; PPV—positive predictive value; NPV—negative predictive value.

**Table 2:** The histopathological types of cancer detected as MFMCC (percentage values of the total incidence).

CNB	All Occurrences	Neoplastic Lesions of MFMCC Nature	MG	CESM	MR	HP
Infiltrating duct carcinoma	2 (3.33%)	1/2 (50.00%)	1 (1.67%)	1 (1.67%)	1 (1.67%)	1 (1.67%)
Invasive lobular carcinoma	45 (75.00%)	24/45 (53.33%)	13 (21.67%)	22 (36.67%)	23 (38.33%)	24 (40.00%)
Special subtype	2 (3.33%)	2/2 (100.00%)	1 (1.67%)	2 (3.33%)	2 (3.33%)	2 (3.33%)
DCIS HG	4 (6.67%)	4/4 (100.00%)	1 (1.67%)	3 (5.00%)	4 (6.67%)	4 (6.67%)
Tubulolobular carcinoma	7 (11.67%)	3/7 (42.86%)	2 (3.33%)	2 (3.33%)	3 (5.00%)	3 (5.00%)
Total	60 (100.00%)	34/60 (56.67%)	18 (30.00%)	30 (50.00%)	33 (55.00%)	34 (56.67%)

Abbreviations: CNB—core-needle biopsy result; MFMCC—multifocality and multicentrality of breast cancer; MG—mammography; CESM—contrast-enhanced spectral mammography; MRI—magnetic resonance imaging; HP—histopathological examination; DCIS HG—ductal carcinoma in situ high grade.

**Table 3:** Changes in surgery extent upon identification of MFMCC in CESM and MRI.

Types of Surgeries	Planned Surgeries Based on MG	Surgeries following MFMCC Visualization in CESM and MRI	Numberof Changes in Surgery Extent into Different Mastectomies	Local Radicalization in the Group of Patients on Conserving Treatment with MFMCC
Different mastectomies	16	26	0	0
WLE + ALND	10	6	4	0
WLE	3	2	1	0
WLE + SLNB	31	26	7	1
In total	60	60	12	1

Abbreviations: MFMCC—multifocality and multicentrality of breast cancer; MG—mammography; CESM—contrast-enhanced spectral mammography; MRI—magnetic resonance imaging; WLE—wide local excision; ALND—axillary lymph node dissection; SLNB—sentinel lymph node biopsy.

## Data Availability

Search results are available from the authors.
